# The Influence of Selected Insecticides on the Oxidative Response of *Atta sexdens* (Myrmicinae, Attini) Workers

**DOI:** 10.1007/s13744-023-01077-7

**Published:** 2023-09-01

**Authors:** Silvana Beani Poiani, Pavel Dobeš, Martin Kunc, Mayara Cristina Pereira, Odair Correa Bueno, Pavel Hyršl

**Affiliations:** 1 Department of Experimental Biology, Faculty of Science, Masaryk University, Brno, Czech Republic; 2 Institute of Biosciences, Center for the Study of Social Insects, Sao Paulo State University UNESP, Sao Paulo, Brazil

**Keywords:** Antioxidant, Flavonoid, Fipronil, Magnesium complex, Sulfluramid

## Abstract

Reactive oxygen species (ROS) are generated as products of normal cellular metabolic activities; however, the use of pesticides to control leafcutter ants leads to unbalanced ROS production. We evaluated the effects of two insecticides (fipronil, sulfluramid) and metallic insecticide complex (magnesium complex [Mg(hesp)2(phen)] (1)) on the superoxide dismutase (SOD), glutathione (GSH) and the overall antioxidant capacity using two different methodologies: total radical-trapping potential (TRAP) and oxygen radical absorbance capacity (ORAC). Media workers of *Atta sexdens* (C. Linnaeus) were exposed to the insecticides for 24 h, 48 h, 72 h and 96 h before their fat bodies were dissected for analysis. The results showed that although the sulfluramid may cause the production of ROS, its slow action in the organism does not lead to oxidative stress. There is a rise in oxidative stress in workers of leafcutter ants treated with fipronil because SOD significantly increased when compared to the control group. On the other hand, Mg1-complex suppressed both GSH and SOD, indicating that the immune system may be affected by Mg1-complex, which has a delayed activity ideal for its use in chemical pest control. Both TRAP and ORAC evaluated total antioxidant capacities; however, ORAC proved to be a more sensitive method. In conclusion, the Mg1-complex is a new compound that should be further investigated as a potential replacement for fipronil and sulfluramid in pest control.

## Introduction

*Atta* species gather pieces of fresh flowers and leaves, attacking many kinds of vegetation, including crop plants, being considered a serious economic pest (Hölldobler and Wilson 1990). The most common and large-scale approach to control leafcutter ants is based on chemical control (Della Lucia et al. 1993).

Toxic baits to leaf-cutting ants containing attractants when sulfluramid and fipronil are the main compounds have widespread use (Forti et al. 1993; Boaretto and Forti 1997). The harvested baits are taken into the nest, thus contaminating the fungus garden and allegedly also the workers when they are handling the fungus, which causes their eventual death within 4 to 5 days (Gallo et al. 1988; Della Lucia et al. 1993). Sulfluramid has largely replaced dodecachlor in toxic baits, although fipronil and a few other compounds are also employed (Boaretto and Forti 1997; Antunes et al. 2000; Montoya-Lerma et al. 2012). However, these compounds’ mechanisms of colony suppression are poorly known.

The production and degradation of sulfluramid involve perfluorooctanesulfonate (PFOS), a substance that is toxic to other insects (Bots et al. 2010), fish (Taniyasu et al. 2003), birds (Verreault et al. 2005; Olivero-Verbel et al. 2006) and mammals (Fan et al. 2005; Luebker et al. 2005). As an effective alternative to the use of sulfluramid, a metallic insecticide complex with lower ecological impact was developed (Bonomo et al. 2019, 2020; Sachi et al. 2021). The magnesium complex [Mg(hesp)2(phen)] (1) (referred here as Mg1-complex) is formed by hesperidin (hesp) and 1,10′-phenanthroline (phen). Contrary to fipronil and sulfluramid, Mg1-complex is hydrosoluble, and it does not require solvents such as acetone and oil commonly used for fipronil and sulfluramid (Oliveira et al. 2013). Mg1-complex was tested in workers of *Atta sexdens *(C. Linnaeus) and has been proved to control them in bioassays (Oliveira 2012).

In general, once the insecticide penetrates the individuals, it overstimulates the production of reactive oxygen species (ROS) (Bhattacharyya et al. 2014). The bodies of insects contain a complex of antioxidants and detoxifying enzymes that act to eliminate ROS (Felton and Summers 1995; Kodrík et al. 2015).

The essential components of the antioxidant system are enzymatic and non-enzymatic antioxidants (Felton and Summers 1995; Arrigoni and De Tullio 2002; Tabrez and Ahmad 2011). Glutathione (GSH) is a vital intracellular and extracellular protective non-enzymatic antioxidant. It plays a variety of crucial roles in the control of signalling processes, detoxifying xenobiotics and heavy metals, as well as other functions (Zitka et al. 2012). Superoxide dismutase (SOD) is one of the essential, potent antioxidant enzymes that scavenge for oxygen free radicals such as superoxide anion (O_2_^•^) and eventually converts it into hydrogen peroxide (H_2_O_2_). The H_2_O_2_ molecule is the substrate for catalases (Khessiba et al. 2005), which neutralize hydrogen peroxide to water and oxygen. A broader view of the total antioxidant status of tissue can be achieved. The total radical-trapping potential (TRAP) and oxygen radical absorbance capacity (ORAC) are methods that embrace different aspects of the antioxidant action on the organism. Those methods measure the ability of antioxidants to scavenge peroxyl radicals via hydrogen atom transfer. These radicals are physiologically the most important ones, and the hydrogen atom transfer is the most physiologically relevant mechanism of antioxidant action (Denev et al. 2014).

Antioxidants are produced in various tissues of insects, such as the fat body. Most of the insect’s intermediary metabolism takes place in this organ, including lipid and carbohydrate metabolism, protein synthesis and amino acid and nitrogen metabolism (Arrese and Soulages 2010). In addition to its role related to storage and utilization of nutrients, the fat body is also an endocrine organ (Hoshizaki 2005), contributes to the insect immune system by releasing effector molecules into the hemolymph (Hoshizaki 2005; Lemaitre and Hoffmann 2007) and participates in detoxification (Arrese and Soulages 2010; Chapman 2012). The insect’s fat bodies are multifunctional organs. In this sense, fat bodies are highly subjected to physiological changes when exposed to xenobiotics and then considered a model for toxicity research. It is expected that any disturbance within this organ may vitally affect an insect’s physiology and behaviour. Changes in activities of antioxidants induced by oxidative stress have been studied in invertebrate tissues (Büyükgüzel et al. 2010; Panzarino et al. 2016), including insect fat body (Hyršl et al. 2007). However, no previous research was performed to evaluate how the antioxidant system behaves when influenced by insecticides in workers of *A. sexdens*.

There is an increasing concern about the ecological effect of the use of insecticides. Thus, it is fundamental to understand the processes involved in the physiological responses of ants aiming at a chemical control, which will be more efficient and less hazardous to the environment. This study aimed to evaluate and to compare the influence of traditional insecticides such as fipronil and sulfluramid, and a new metallic insecticide (Mg1-complex) on SOD, GSH and total antioxidant capacity in the fat body cells of *A. sexdens* workers. We hypothesize that the studied insecticides alter the antioxidant balance in the fat body of worker ants, causing oxidative stress.

## Material and methods

### Ants

Media workers of *A. sexdens* were collected from the laboratory nest kept at Center for the Study of Social Insects (CEIS), Institute of Biosciences, Sao Paulo State University. The nest was daily supplied with leaves of *Eucalyptus* sp., oat seeds, and occasionally with leaves of other plants such as *Hibiscus* sp., *Ligustrum* sp. or rose petals.

**Fig. 1:**
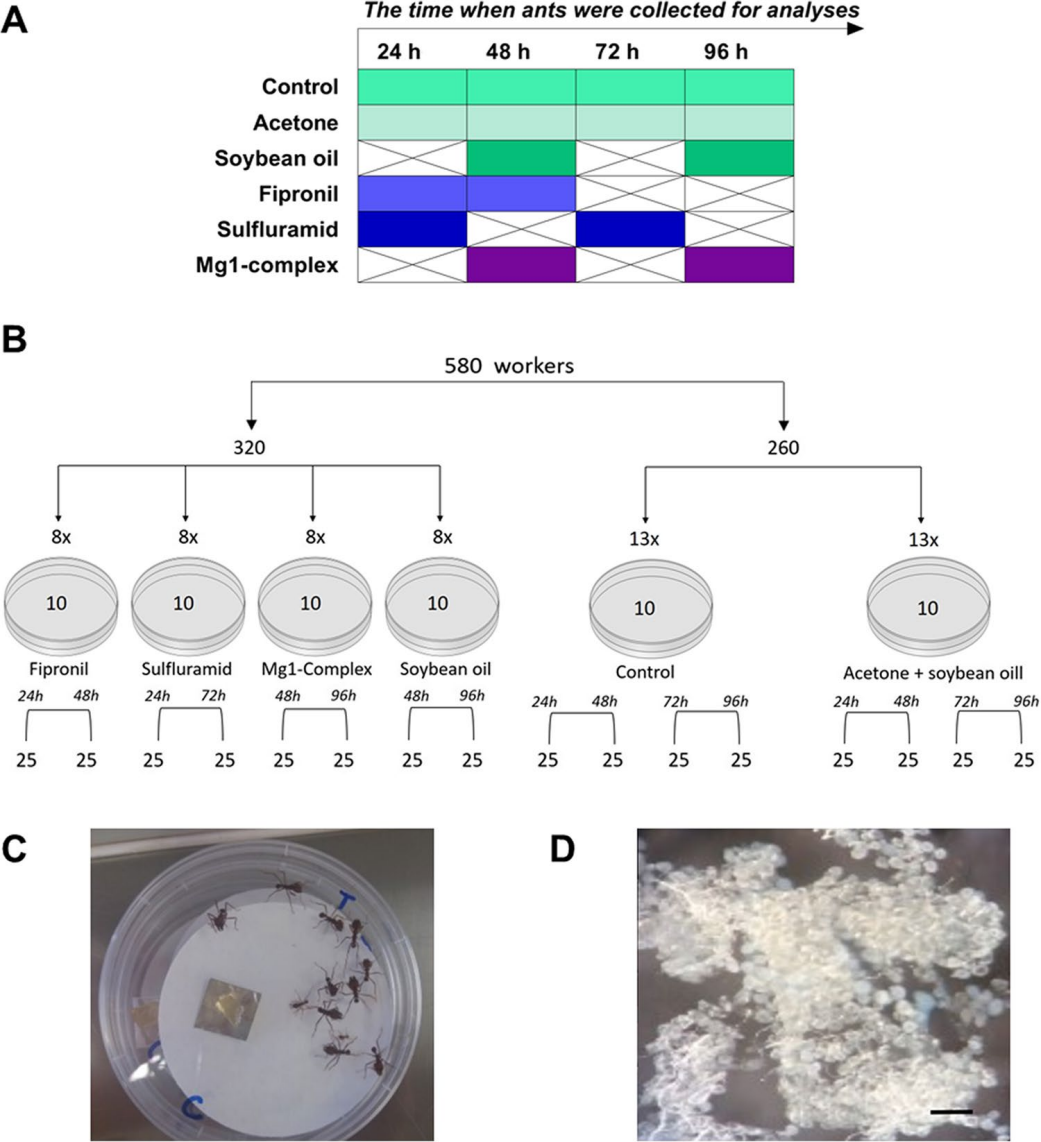
**A** Timetable showing the design of insecticide treatment and sample collection in the control and experimental groups. The mode of action for each tested insecticide was considered in the design of sampling intervals. The colour indicates the time points when samples were collected for analysis. **B** Scheme of bioassay design showing the experimental groups and number of workers used in the study. **C** Petri dish containing workers fed on the artificial food. **D** Fat body dissected after exposition to an insecticide; scale bar corresponds to 50 μm

A total of 580 media workers were collected from the nest for the bioassays. From that amount, 260 were divided into two groups (control and solvent) containing 130 workers each. The other 320 were divided into four groups (fipronil, sulfluramid, Mg1-complex and soybean oil) containing 80 workers each. The workers were placed in Petri dishes (10 per each Petri dish) (Fig. 1).

In total, six groups of ants were analyzed: *control* (without solvent and insecticides); acetone + soybean oil (the solvent, further referred to as “acetone”); *sulfluramid* diluted in solvent (0.005%; a respiratory poison insecticide); *fipronil* diluted in solvent (0.001%; a neurotoxic insecticide); *Mg1-complex*, magnesium complex [Mg(hesp)2(phen)] (1) (2 mg/mL; a metallic insecticide administrated without solvent) and *soybean oil* without acetone (2%). The ants were exposed to insecticides orally by ingestion of an artificial diet containing insecticides, which was offered ad libitum and replenished daily (Bueno et al. 1997). Previous bioassays in our laboratory already established the median lethal time concentration of each insecticide. The ants were collected at different times (Fig. 1A) for analysis because the insecticides have different times of action in the organism. The fat bodies of 25 workers of each group and time (Fig. 1B–D) were dissected and placed into the appropriate buffer containing a few phenylthiourea (PTU) crystals to avoid melanisation. Subsequently, in the collection, the samples were stored in a freezer at − 20 °C till analyses (Hyršl et al. 2007).

### Glutathione (GSH) activity assay

The fat bodies of workers were 16 × diluted in 125 mM Na-phosphate buffer containing 6.3 mM Na-EDTA (pH 7.5) and homogenized. After, the samples were centrifuged at 10,000 g for 10 min. The supernatants were transferred into another set of tubes.

In a 96-well transparent plate, it was added 50 μL of samples (or standard), 175 μL of 0.3 mM NADPH (Sigma-Aldrich, St. Louis, MO, USA) and 10 μL of glutathione reductase (50 units of glutathione reductase/mL, Sigma-Aldrich, St. Louis, MO, USA). The microtiter plate was incubated for 10 min at 37 °C and then 25 μL of 6 mM DTNB (Sigma-Aldrich, St. Louis, MO, USA) was added. Absorbance was read at 412 nm for 30 min with 5-min intervals using multi-mode microplate reader Sense (Hidex, Finland). GSH (Sigma-Aldrich, St. Louis, MO, USA) standard dilutions (3.13, 1.56, 0.78, 0.39 and 0.19 μM) were used to calculate GSH content from linear regression. After, the values were multiplied by the dilution factor. The measurements were replicated four times.

### Superoxide dismutase (SOD)

SOD activity was determined based on the ability of the enzyme to inhibit the autoxidation of pyrogallol. The autoxidation of pyrogallol was measured in the presence of EDTA and pH 8.2. One unit (1U) of SOD activity is defined as the amount of enzyme required to inhibit the autoxidation of pyrogallol by 50% per min per millilitre of the assay mixture. The principle of this method is based on the competition between the pyrogallol autoxidation by O_2_^•^¯ and the dismutation of this radical by SOD (Magnani et al. 2000). For total SOD activity, the fat bodies were 4 × diluted in Tris–EDTA buffer (50 mM Tris, 1 mM EDTA) (pH 8.2). The samples were centrifuged at 10,000 g for 10 min at 4 °C. Total SOD activity was determined according to the method of Marklund and Marklund (1974); specifically, in a 96-well plate, it was added 20 μL of supernatant from samples and 180 μL 0.2 mM pyrogallol (Sigma-Aldrich, St. Louis, MO, USA). The absorbance was measured at 420 nm using the multi-mode microplate reader Sense (Hidex, Finland) for 20 min. The measurements were replicated four times. SOD content was calculated from the calibration curve drawn from serial dilutions (0.3, 0.15, 0.074, 0.037, 0.018, 0.009 and 0.004 U) of bovine superoxide dismutase (Sigma-Aldrich, St. Louis, MO, USA). One unit (U) total SOD activity was calculated as the amount of the enzyme causing 50% inhibition of pyrogallol auto-oxidation.

Before total SOD activity calculation, the percentage of inhibition of SOD was assessed using the formula:

1 $$\mathrm{Pi}=100\ast\left(\left[\Delta\mathrm{OD}\;\mathrm{Tris}-\mathrm{EDTA}\right]-\left[\Delta\mathrm{OD}\;\mathrm{of}\;\mathrm{sample}\;\mathrm{or}\;\mathrm{standard}\right]/\left[\Delta\mathrm{OD}\;\mathrm{Tris}-\mathrm{EDTA}\right]\right)$$

where:

- Pi = percentage of the inhibition;
- ΔOD = the change of optical density per min; the slope value from linear regression of each sample/standard solution.

The formula for total SOD activity calculation expressed as units per millilitre (U/mL) was:

2 $$\left[\mathrm{SOD\; activity}\right]=\left(\mathrm{Pi}*\mathrm{Vt}*\mathrm{D}\right)/\left(50*\mathrm{Vs}\right)$$

where:

- Pi = percentage of the inhibition;
- Vt = the total volume of solution (200 μL in the present study);
- D = the dilution factor used (4 × in the present study);
- 50 = 50% inhibition of pyrogallol autoxidation (1 U of SOD activity);
- Vs = the sample volume used (20 μL).

### Total radical-trapping antioxidant parameter (TRAP)

One of the most employed procedures to evaluate the antioxidant status of biological tissue is the total radical-trapping potential (TRAP). The method was developed by Wayner et al. (1985) and the methodology is based on measurements of induction times in the oxidation of a lipid dispersion exposed to a free radical source with a constant (and known) rate of free radical. The 2,2′-Azo-bis(2-amidinopropane) (ABAP) is used as the free radical source (Lissi et al. 1995), and those radicals react directly with luminol or are trapped by antioxidants. Luminol-enhanced chemiluminescence (CL) was used to follow the peroxyl radical reaction, and the principle was described previously in (Cizova et al. 2004). Radicals are derived from the thermal decomposition of ABAP and the CL signal is driven by the production of light after luminol excitation. The TRAP value is determined from the duration of the period during which the sample quenched the CL signal due to the present antioxidants (Denev et al. 2014). Four different standard concentrations (0.5, 1.0, 1.5 and 2.0 nmol/mL) of the standard antioxidant Trolox (6-hydroxy-2,5,7,8,-tetramethylchroman-2-carboxylic acid; Sigma-Aldrich, St. Louis, MO, USA) were prepared for calibration curve.

For TRAP measurement, the same amount of collected fat bodies from all groups was 7 × diluted in PBS and homogenized. After, 160 μL PBS, 16.7 μL luminol (10 mM; Sigma-Aldrich, St. Louis, MO, USA) and 20 μL of the sample were added to the non-transparent white microtiter plate. The plate was incubated at 37 °C for 10 min. Then, 16.7 μL of cold 400 mM ABAP (Sigma-Aldrich, St. Louis, MO, USA) was added. Luminescence was measured for 1.5 h at 37 °C using the luminometer Chameleon V (Hidex, Finland). The measurements were replicated three times.

The total antioxidant capacity was calculated from the linear regression curve prepared from serial dilution of standard antioxidant Trolox. After, the values were multiplied by the dilution of samples used. Data were expressed as mM Trolox equivalents (TE).

### Oxygen radical absorbance capacity (ORAC)

ORAC is one of the most used methods to evaluate the capacity of antioxidants to inhibit the bleaching of a target molecule (probe) induced by peroxyl radicals. ORAC assay originally used phycoerythrin as a probe (Cao et al. 1993). However, fluorescein (3’,6’-dihydroxyspiro[isobenzofuran-1(3H),9’-[9H]xanthen]-3-one) can be used as the target molecule of oxygen radicals (Ou et al. 2001; Dávalos et al. 2004). Usually, Trolox and ABAP are employed as reference antioxidant and peroxyl radical source, respectively (Niki 1990). ABAP is used to produce peroxyl radicals that react with fluorescein giving non-fluorescent products. The addition of an antioxidant delay the fluorescence decay. The quantification of the antioxidant capacity is carried out from the net integrated areas under the fluorescence decay curves and accounts lag time, initial rate and total extent of inhibition in a single value. The results are compared to those of a reference antioxidant, usually Trolox (Bentayeb et al. 2014).

For ORAC analysis, the fat bodies were diluted 2000 × . In a 96-well dark plate, it was added 10 μL of samples or Trolox (Sigma-Aldrich, St. Louis, MO, USA), 170 μL fluorescein (10 nM; Sigma-Aldrich, St. Louis, MO, USA) and incubated for 30 min at 37 °C. After, 20 μL ABAP (0.1 M; Sigma-Aldrich, St. Louis, MO, USA) was added to each well. Standard Trolox dilutions (0.03, 0.06, 0.125, 0.25, 0.5 and 1 mM) were performed for calibration curve. Fluorescence was measured using an excitation wavelength of 485 nm and an emission of 528 nm for 2 h using a multi-mode microplate reader Sense (Hidex, Finland).

The area under the curve (AUC) of all samples/Trolox standards/blank was calculated, where blank is the PBS only. After, the Net AUC values were calculated for each standard Trolox and sample according to the formula:

3 $$\left[\mathrm{Net}\;\mathrm{AUC}\right]=\left[\mathrm{AUC}\;\mathrm{Trolox}\;\mathrm{or}\;\mathrm{sample}\right]-\left[\mathrm{AUC}\;\mathrm{blank}\right]$$

The Net AUC values of Trolox were plotted against the Trolox concentrations, and the linearity of the standard curve was calculated. ORAC values of samples were calculated using the linear regression equation. The values obtained were multiplied by the dilution used. Data were expressed as mM Trolox equivalents (TE).

### Statistical analyses

All data were assessed for normality and homogeneity of variance prior to one-way ANOVA or Student *t*-test. Tukey’s post hoc test was used to determine which means differ from the rest in multiple comparisons. All analyses were performed using Prism 7.0 software, and *P*-values < 0.05 were considered significant. Mean and standard deviation are present in all figures.

## Results

### GSH

**Fig. 2:**
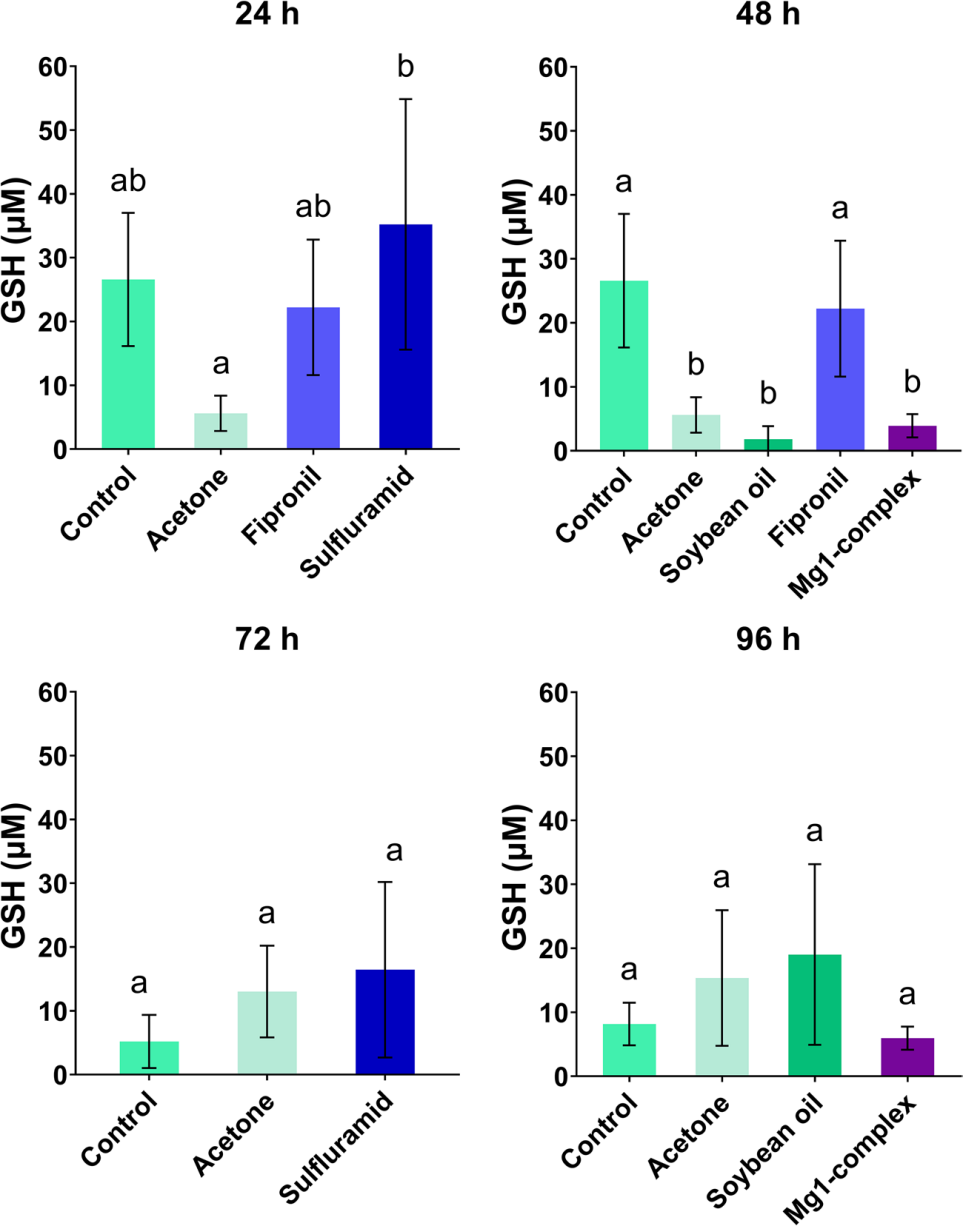
Glutathione (GSH) concentration analyzed at 24 h, 48 h, 72 h and 96 h in the control and insecticide-treated groups. The results are shown as mean ± standard deviation; the results of multiple comparisons (Tukey’s post hoc test) are marked with letters above columns. The data bars labelled with a different letter are significantly different at the 5% level (*P* < 0.05)

Statistical analysis revealed that there were groups that statistically differ from each other at 24 h and 48 h (ANOVA, *P* < 0,05). At 24 h, GSH content in the group exposed to sulfluramid was higher than in acetone group (Tukey’s test, *P* = 0.02) (Fig. 2). At 48 h, the control group has a higher amount of GSH in comparison to acetone (Tukey’s test, *P* = 0.001), Mg1-complex (Tukey’s test, *P* = 0.002) and soybean oil (Tukey’s test, *P* = 0.0008) groups. Also, fipronil statistically differs from acetone (Tukey’s test, *P* = 0.0001), Mg1-complex (Tukey’s test, *P* = 0.0003) and soybean oil (Tukey’s test, *P* < 0.0001) (Fig. 2). There was no significant difference among groups at 72 h and 96 h (ANOVA, *P* > 0.05).

### SOD

**Fig. 3:**
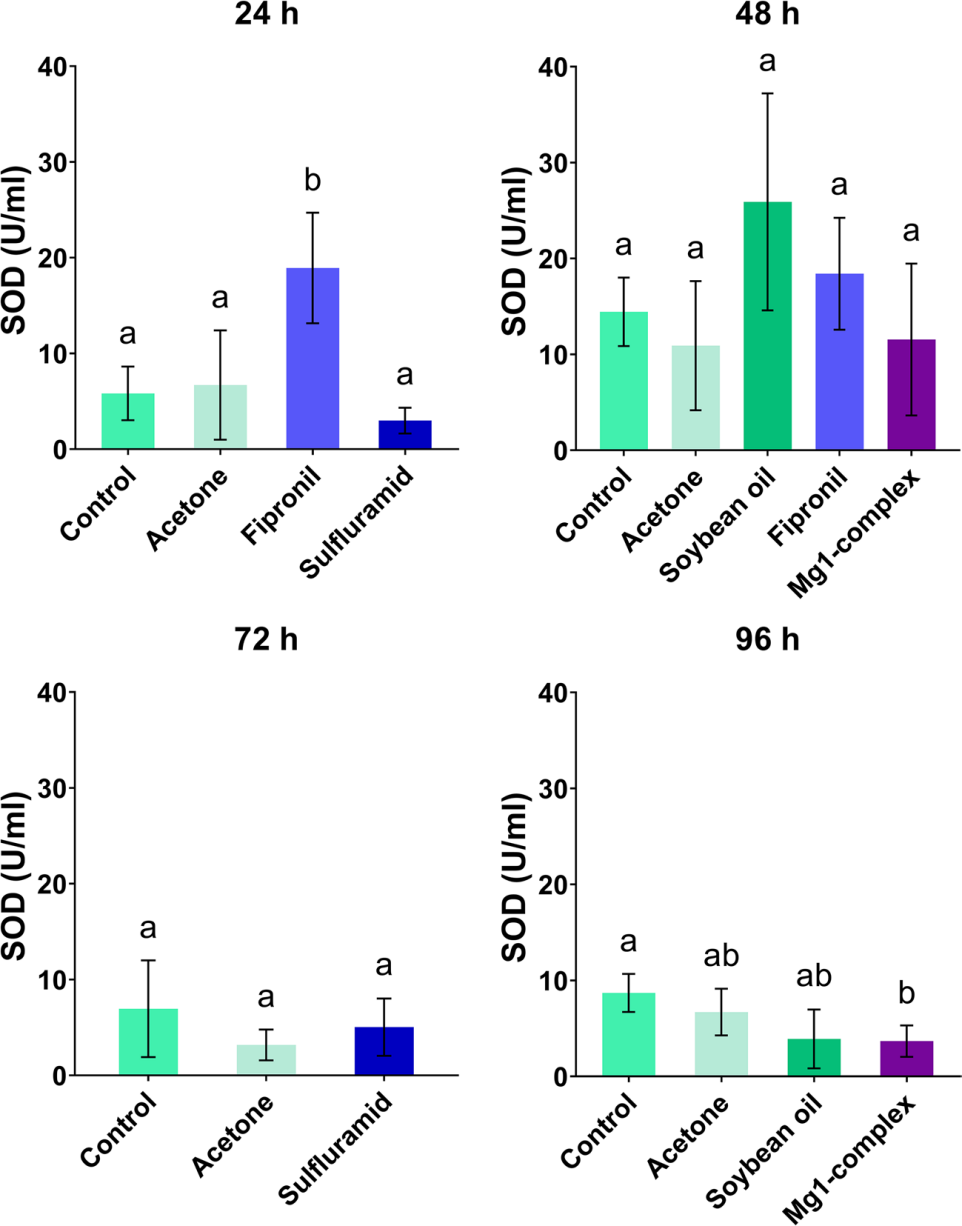
The activity of superoxide dismutase (SOD) at 24 h, 48 h, 72 h and 96 h in the control and insecticide-treated groups. The results are shown as mean ± standard deviation; the results of multiple comparisons (Tukey’s post hoc test) are marked with letters. The data bars labelled with a different letter are significantly different at the 5% level (*P* < 0.05)

Statistical analysis indicates that the amount of SOD varied among the groups at 24 h and 96 h (ANOVA, *P* < 0.05). There is a significant increase of SOD in fipronil at 24 h in comparison with control (Tukey’s test, *P* = 0.0052), while Mg1-complex caused a significant decrease of SOD compared to control (Tukey’s test, *P* = 0.0450) at 96 h. There was no significant difference among the tested groups at 48 h and 72 h (ANOVA, *P* > 0.05) (Fig. 3).

### Total antioxidant capacity measured by TRAP

**Fig. 4:**
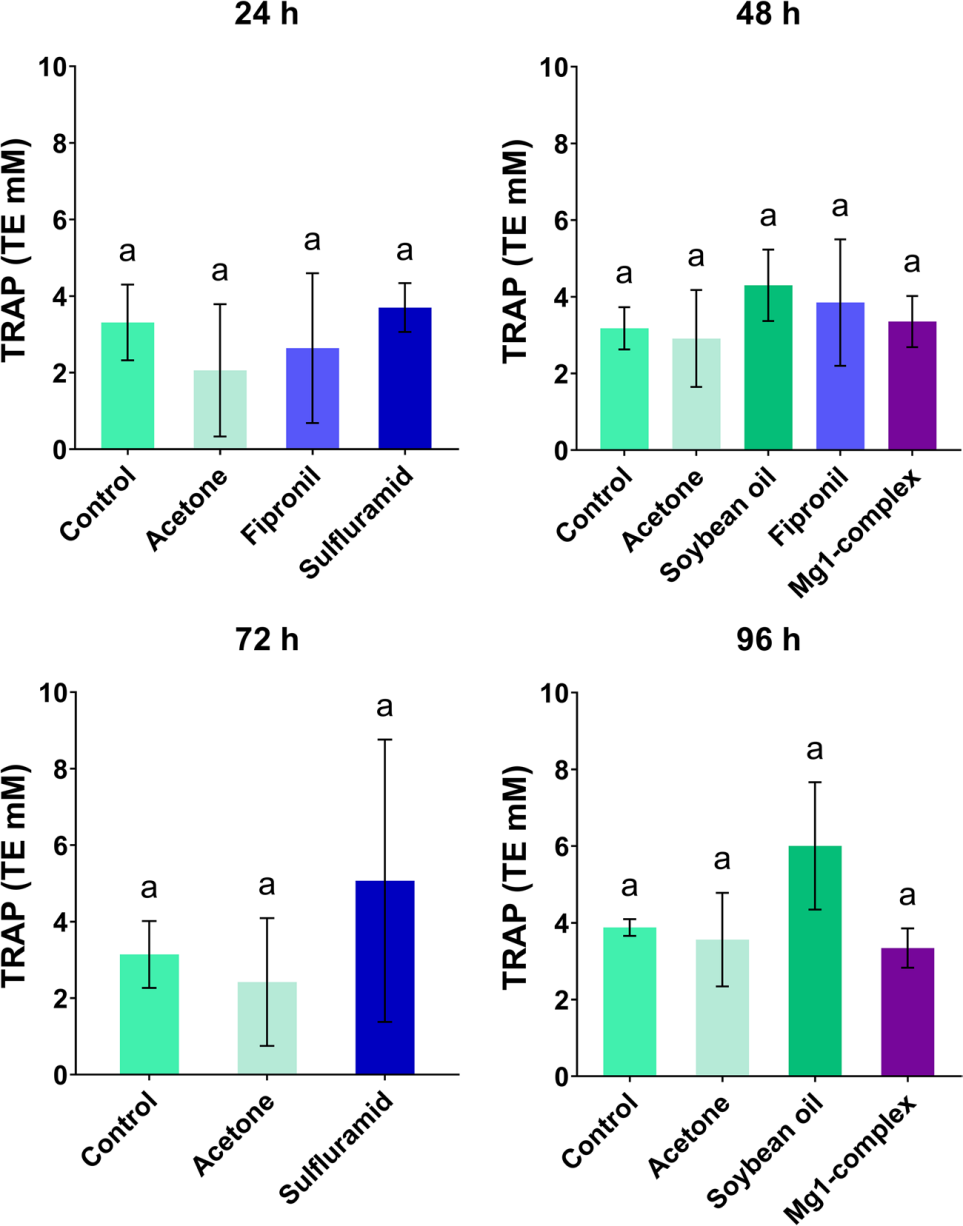
Total radical-trapping antioxidant parameter (TRAP). Means and standard deviation of the quantity of total antioxidant capacity with time among the analyzed groups at 24 h, 48 h, 72 h and 96 h. The results of multiple comparisons (Tukey’s post hoc test) are marked with letters. The data bars labelled with a different letter are significantly different at the 5% level (*P* < 0.05). No significant effect of insecticides on total antioxidant capacity was observed (ANOVA, *P* ˃ 0.05)

Total radical-trapping antioxidant potential (TRAP) of dissected fat body tissue was measured to evaluate potential changes in overall antioxidant level caused by the tested insecticides. Statistical analysis revealed no significant effect of insecticides on total antioxidant capacity (Fig. 4; ANOVA, *P* ˃ 0.05). For instance, an antioxidant capacity in the control group, fipronil and Mg1-complex was 3.18 ± 0.55 mM, 3.85 ± 1.65 mM and 3.35 ± 0.69 mM of the standard antioxidant trolox, respectively, 48 h after the treatment. The same total level of antioxidants was observed in all tested insecticides and sampling time points (Fig. 4; ANOVA, *P* ˃ 0.05).

### Total antioxidant capacity measured by ORAC

**Fig. 5:**
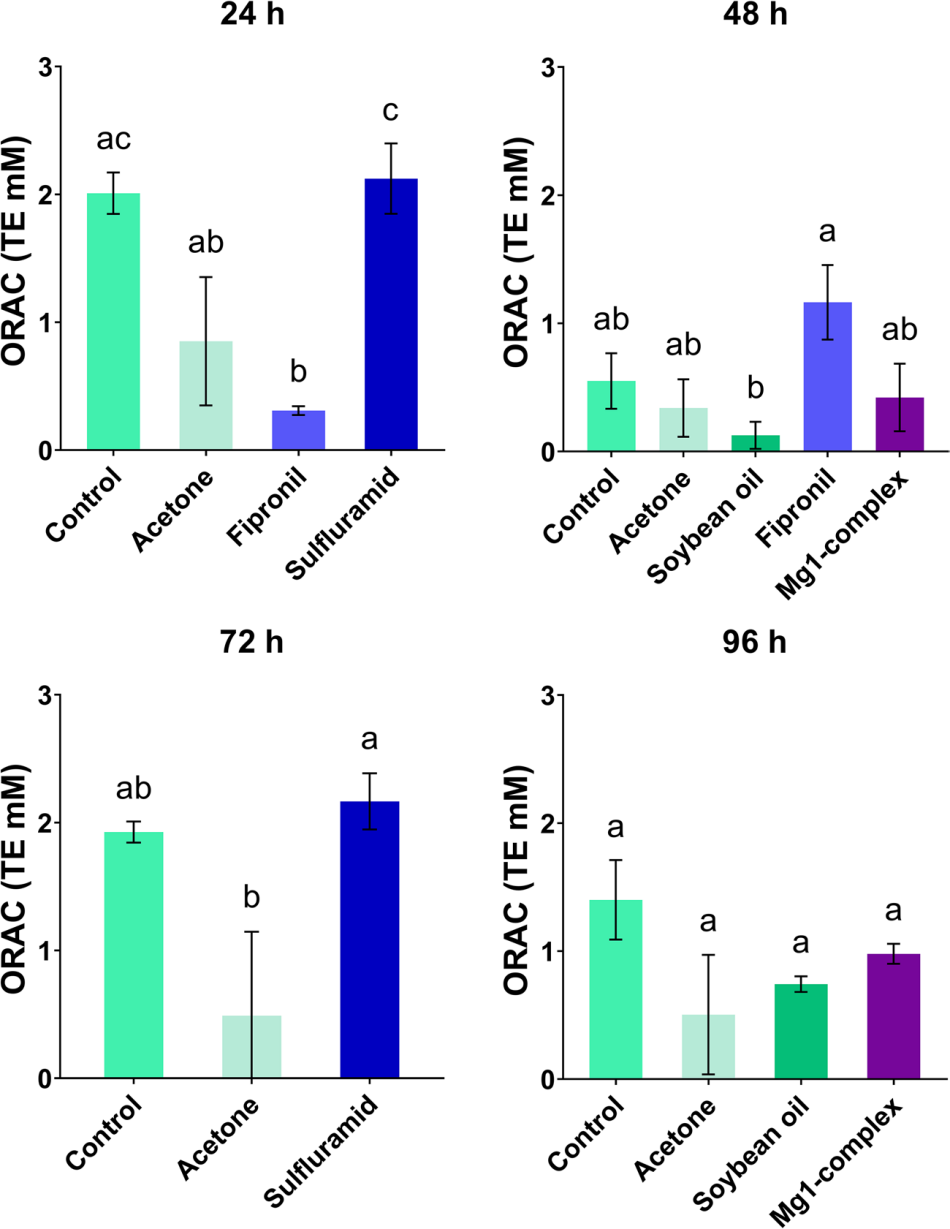
The antioxidant capacity measured as ORAC and its changes with time among the analyzed groups at 24 h, 48 h, 72 h and 96 h. The results are shown as mean ± standard deviation; the results of multiple comparisons (Tukey’s post hoc test) are marked with letters. The data bars labelled with a different letter are significantly different at the 5% level (*P* < 0.05). The capacity of antioxidants is expressed in mol Trolox equivalent (TE)

ORAC analysis also measured the total capacity of antioxidants. Among the groups that were collected for analysis at 24 h, some treatments differ from others (ANOVA, *P* < 0.05): The control group statistically differed from fipronil (Tukey’s test, *P* = 0.0158); also, sulfluramid differs from acetone (Tukey’s test, *P* = 0.0427) and from fipronil (Tukey’s test, *P* = 0.0125) (Fig. 5). At 48 h (ANOVA, *P* < 0.05), fipronil and soybean oil were statistically different (Tukey’s test, *P* = 0.0312). At 72 h (ANOVA, *P* < 0.05), acetone is statistically different from control (Tukey’s test, *P* = 0.0113) and from sulfluramid (Tukey’s test, *P* = 0.0026). There was no significant difference among groups at 96 h (ANOVA, *P* > 0.05) (Fig. 5).

## Discussion

The most common insecticides used to control agricultural pests are toxic for non-target animals and are also hazardous to the environment. The control of leafcutter ants is an economic issue that is leading researchers to try to find new chemicals able to control those ants with less damage to the environment.

Traditional insecticides and the metallic insecticide complex were used for comparison of their actions on the antioxidant system of *A. sexdens* workers. The results showed that the dynamic of antioxidants changes in the fat body of workers of *A. sexdens*, depending on which insecticide the workers were exposed to and on the exposure time. In general, these results agree with earlier reports that insecticides are responsible for causing oxidative stress in insects (Üner et al. 2006; Lushchak 2012).

Sulfluramid is one of the most often insecticide used for leafcutter control, and there was no difference when compared to the control group for GSH, SOD or total antioxidant capacity. The level of antioxidants in fat bodies seems not to be affected or were not detected in 24 h and 72 h of sulfluramid exposition. It is known that sulfluramid acts as respiratory poison, demonstrating its lethal delayed action, as observed in the *Camponotus pennsylvanicus* (C. DeGeer) ant (Reid and Klotz 1992). The sulfluramid, when present in the body, is metabolized to perfluorooctane sulfonamide (DESFA), which operates in the oxidative phosphorylation process (aerobic respiration), interrupting the production of ATP in mitochondria, becoming lethal to the insect (Schnellmann and Manning 1990). Although the sulfluramid may cause the production of ROS, we can speculate that its slow action in the organism does not lead to an oxidative stress condition. The characteristic symptom of intoxication by sulfluramid in *Atta* spp. workers is manifested by slow movements and a significant decrease in aggressiveness due to the energy reduction of their organism (Schnellmann and Manning 1990).

Fipronil is among the new chemical groups used to control leaf-cutting ants. It belongs to the chemical group of phenyl pyrazoles that act on the central nervous system, specifically in the gamma-aminobutyric acid (GABA) system (Tomlin 2000). According to our results, GSH of groups exposed to fipronil (24 h and 48 h) did not differs statistically from the control. On the other hand, the level of SOD statistically increased at 24 h in comparison with control. Fipronil is known to present quick action in the organism. It seems that the exposition time could be related to GSH not being affected. The GSH and its oxidized form GSSG are part of the dynamic system in which the reduced form can be quickly regenerated through enzymatic antioxidants. For further investigations, analysing GSH at shorter time intervals (e.g. 5 h, 10 h, 15 h) instead 24 h would be interesting. Antioxidant enzymes such as SOD are of vital importance in an organisms’ defence against oxidative stress (McCord and Fridovich 1969; Mamidala et al. 2011) and it has been associated with insecticide toxicity in insects (Landa et al. 1991), frogs (Czarniewska et al. 2003) and also in freshwater clams (Conners 2004). Our results suggest a rise in oxidative stress in workers of *A. sexdens* treated with fipronil. *Apis cerana* (J. C. Fabricius) and *Apis dorsata* (J. C. Fabricius) also showed overexpressed SOD when treated with insecticides (Chakrabarti et al. 2015). According to Akhgari et al. (2003), SOD could be an adaptive response as a protective mechanism to an imbalanced level of free radicals within the body. Although SOD activity increased, the total antioxidant capacity measured by ORAC at 24 h is significantly lower than control. The measure of antioxidant capacity, such as ORAC, considers the cumulative action of all the antioxidants present in body fluids or tissues, thus providing an integrated parameter rather than the simple sum of measurable antioxidants (Ghiselli et al. 2000). The depletion of some antioxidants in the mixture may cause an overall decrease in the antioxidant system, indicating that the fipronil is affecting the redox balance and the defence system at 24 h. There was no significant change observed after 24 h of exposition to fipronil. In the firebug *Pyrrhocoris apterus* (C. Linnaeus), the GSH decreased 3 h after the injection of two different xenobiotics (Krishnan et al. 2007; Večeřa et al. 2007). It may be possible that some changes could occur earlier than 24 h in the fat bodies of workers of *A. sexdens*, and since we analyzed at 24 h, the change was not detected.

Mg1-complex is a recently developed metallic insecticide that deserves more attention. Bioassays performed has shown that Mg1-complex is not a fast-killer and acts slowly in workers of *A. sexdens* (Oliveira 2012). However, its physiological action in workers of *A. sexdens* is not explicit because no experiments have been performed. The present research is the first to evaluate the antioxidant parameters in workers of *A. sexdens* exposed to Mg1-complex. GSH is the major antioxidant and takes part in several protective roles (Masella et al. 2005). At 48 h, there was a significant decrease in GSH level of the Mg1-complex group in comparison with control. The decrease of GSH was expected because it is involved in a variety of detoxifying processes. GSH acts as a cofactor of several detoxifying enzymes against oxidative stress, e.g. glutathione peroxidase, glutathione-S-transferase and others; GSH scavenges hydroxyl radical and singlet oxygen directly, detoxifying hydrogen peroxide and lipid peroxides by the catalytic action of ascorbate peroxidase or glutathione-S-transferase; and GSH can regenerate other antioxidants, such as vitamins C and E, back to their active forms (Krishnan et al. 2009). Different xenobiotics decrease the GSH level in a variety of species: malathion poisoning in humans (Banerjee et al. 1998); firebug *P. apterus* treated with paraquat (Krishnan et al. 2007; Večeřa et al. 2007); poultry birds exposed to xenobiotics (Kumar et al. 2010) and rats exposed to a mixture of synthetic pyrethroids and organophosphate xenobiotics (El-Demerdash 2011). Reduction in the GSH level indicates that detoxification is going on (Ezeji et al. 2012).

As already mentioned above, when discussing fipronil, the SOD level typically increases in organisms under oxidative stress. However, workers exposed to Mg1-complex had significantly lower level of SOD than control at 96 h. Mg1-complex contains hesperidin that has antioxidant activity. Its antioxidant activity increases when it is associated with magnesium (Oliveira et al. 2013). It is not clear how the interaction between hesperidin and SOD can lead to suppression of SOD content. Mg1-complex may downregulate SOD content; however, further experiments with adequate methodology should be performed to clarify the effects of Mg1-complex on SOD. From the results, it can be concluded that Mg1-complex affected the SOD content in a different way observed for other insecticides in the literature (Chakrabarti et al. 2015), and since SOD is suppressed, the immune system may be affected by its low activity capacity.

The acetone group corresponds to the solvent (acetone + soybean oil) used in fipronil and sulfluramid. For GSH at 48 h, both acetone and soybean oil groups differ from control. Since fipronil was not statistically different compared to control at 48 h, it seems that acetone, when used as a vehicle, does not have significant effect on insecticide action, indicating that the soybean oil has an evident effect on GSH depletion. Acetone and soybean oil may have a synergistic effect causing GSH decrease or soybean oil alone is responsible for the GSH depletion since it has been used before as pesticide, herbicide and fungicide (Baker et al. 2018). As a pesticide, it was used in mites, aphids, whiteflies, scales, moths and beetles (Pless et al. 1995; Koona and Njoya 2004; Williamson 2004; Baker et al. 2018), acting by suffocating them. Its effect on GSH content in ants exposed for 48 h shows that soybean oil can cause oxidative stress, playing a key role when used as pesticide vehicle in leafcutter ants. Nutrition stress caused by high-fat diets can exacerbate the ROS production, inducing tissue oxidative damage (Halliwell and Gutteridge 2015, García-Meilán et al. 2023).

The TRAP and ORAC methods embrace different aspects of the antioxidant action and give a broader view of the antioxidant potential of the fat bodies. According to our results, ORAC seems to be more sensitive than TRAP since no differences were detected by TRAP using samples seven times diluted, while ORAC showed statistically significant changes when samples were 2000 × diluted. The sensitivity of both methods is affected by the ratio between the source of radicals (ABAP in this study), chemiluminescent or fluorescent probe (luminol and fluorescein, respectively), and antioxidants present in the sample or standard. In complex fat body samples containing multiple types of antioxidants, the design of ORAC provides a broader range of detection combined with excellent sensitivity in low concentrations of antioxidants (Cao and Prior 1998). Moreover, the antioxidant effect is measured directly against the free radicals that quench fluorescein in the simple reaction, whereas the chemiluminescence curves in TRAP are more complex due to the dynamics of luminol excitation.

The present research contributed to shed light on important antioxidant compounds of workers of *A. sexdens* when exposed to different insecticides. The Mg1-complex is a new compound that should be further investigated to be used as a replacement for fipronil and sulfluramid.

## Data Availability

Data available on request from the authors.
